# Rationale and methods of the MOVI-da10! Study –a cluster-randomized controlled trial of the impact of classroom-based physical activity programs on children’s adiposity, cognition and motor competence

**DOI:** 10.1186/s12889-019-6742-0

**Published:** 2019-04-18

**Authors:** Mairena Sánchez-López, Abel Ruiz-Hermosa, Andrés Redondo-Tébar, María Eugenia Visier-Alfonso, Estela Jimenez-López, Maria Martínez-Andres, Montse Solera-Martínez, Alba Soriano-Cano, Vicente Martínez-Vizcaíno, on behalf of MOVI group, Carlos Berlanga-Macías, Blanca Notario-Pacheco, Ana Díez-Fernández, María Jesús Pardo-Guijarro, Marta Nieto-López, Alberto González-García, Jorge Cañete García-Prieto, Ana Torres-Costoso, Antonio Gracía-Hermoso, Caterina Pesce, Ricardo Cuevas-Campos

**Affiliations:** 1 Universidad de Castilla-La Mancha, Social and Health Care Research Center, C/ Santa Teresa Jornet s/n, 16071 Cuenca, Spain; 2 Universidad de Castilla-La Mancha, Faculty of Education, C/ Ronda de Calatrava 3, 13071 Ciudad Real, Spain; 3 Universidad Autónoma de Chile, Faculty of Medicine, Avenida Pedro de Valdivia, 425, Providencia Talca, Chile

**Keywords:** Active breaks, Classroom-based physical activity, Cognition, Academic performance, Adiposity, Preschooler

## Abstract

**Background:**

Although physical activity (PA) integrated in schools’ classrooms have shown a positive effect on children’s behaviors, its effectiveness on cognitive functions, PA levels and other health variables remains unclear. This article outlines the rationale and methods of two classroom-based PA interventions (MOVI-da10!) on improving adiposity, executive function and motor competence in preschool children.

**Methods:**

A three-arm cluster-randomized controlled trial (RCT) was carried out including eight schools (rural and urban areas) from Cuenca province, Spain. The schools were allocated to one of three groups: MOVI-da10-Enriched! intervention (*n* = 3), MOVI-da10-Standard! intervention, (*n* = 2), and the control group (n = 3). Around 900 children aged 4 to 6 years old were assesed at baseline (September 2017) and at the end (June 2018) of the intervention. The primary outcomes were changes in body fat by bioimpedance, executive function and motor competence. During a school year (from October 2017 to May 2018), children belonging to the MOVI-da10-Enriched! group performed enriched PA integrated into the academic curriculum including two active breaks lasting 10 min, 5 days/week. The children belonging to the MOVI-da10-Standard! group performed PA breaks (with low cognitive demand, where curricular contents were not reinforced) including two active breaks lasting 10 min, 5 days/week. In the control group, regular PA continued.

**Discussion:**

To our knowledge, MOVI-da10! is the first RCT to examine the effectiveness of two programs (enriched PA integrated into the academic curriculum and PA breaks only) versus a control group on improving adiposity, executive function and motor competence in preschool children.

**Trial registration:**

NCT03236363 (clinicaltrials.gov), 31st July 2017.

## Background

Physical activity (PA) has been positively associated with academic performance and health in children [1]. Unfortunately, in Spain, only 30.4% of boys and 12.3% of girls (2 to 10 years old) meet the current health recommendations of accumulating at least 60 min of moderate-to-vigorous intensity PA every day [2], and compliance figures decline with increasing age [3].

Additionally, although obesity is the result of a complex multifactorial combination of genetic and environmental factors, previous studies have described that physical inactivity plays a crucial role in the development of childhood obesity [4]. In Spain, several studies have shown that the prevalence of overweight/obesity is around 21% in children aged 4 to 6 years old [5, 6], which is similar to that reported in other regions of the world [7]. As in adults, obesity in children and adolescents has been associated with adverse health consequences, including hypercholesterolemia, hypertension and bone diseases [8]. Besides, obesity has also been negatively associated with academic performance and health related quality of life and worse mental health [9–11]. Conversely, physical fitness has been identified as an important health marker in children [12].

Although schools are considered to be a good setting for the promotion of children’s PA [13, 14], adding PA to the school schedule can sometimes be complicated due to the time constraints often imposed by key learning areas. Thus, classroom-based PA constitutes a promising strategy for children to be active at school [15], which consists of implementing PA breaks, at any level of intensity, guided by the teacher during normal classroom time. Although several approaches have been described to integrate PA in the classroom, two of them are most commonly implemented: (i) perform physically active lessons or PA breaks while learning or reinforcing academic contents of different subjects; and (ii) perform PA breaks without learning or reinforcing academic contents. Although both strategies separately have been shown to be effective in improving classroom behaviours (e.g. on-task behaviour) [16], there is not enough evidence to conclude which of the two approaches may be more effective in improving academic performance in children, since only one study has compared the effect of PA breaks integrating curriculum contents, PA breaks only and traditional instruction on math performance. After an eight-week intervention, the children in the group that participated in the PA breaks integrating curriculum contents showed greater improvements in math than children in the other groups [17].

Regarding the most effective type of exercise in improving cognitive performance, enriched PA (that requires great cognitive demands, such as perceptive motor activities) seems to result in greater improvements in cognitive processes and motor competence than standardized and repetitive exercises (such as walking or running) [18–20]. In this regard, a study that compared PA breaks with different cognitive demands concluded that only highly cognitive demanding breaks resulted in a significant increase in attention levels [21].

The present study therefore reports on the methods and rationale of a cluster randomized-controlled trial (RCT) aimed at assessing the effectiveness of two types of PA break interventions on improving adiposity and cognition by increasing cardiorespiratory fitness in schoolchildren compared to a control group: one including PA breaks only (MOVI-da10-Standard!) and another including enriched PA breaks (MOVI-da10-Enriched!). A secondary objective was to evaluate the effectiveness of these programs on increasing PA, and improving health related quality of life and motor competence.

## Methods/design

### Study design

**Fig. 1:**
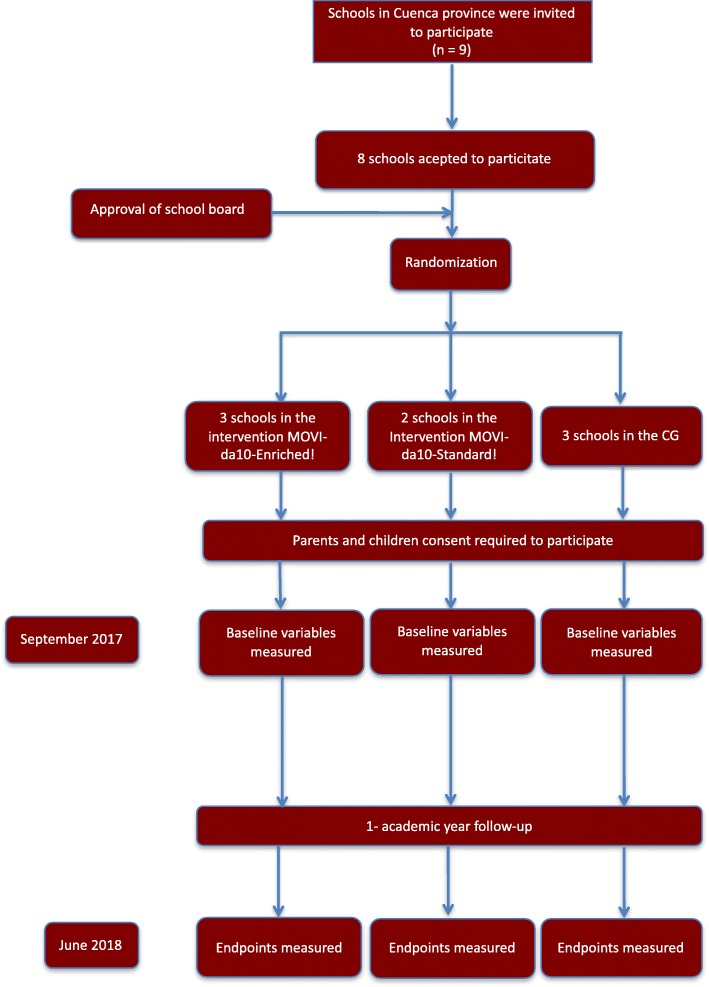
Flow chart of trial participants

This study was designed as an RCT with three arms. Nine schools located in the various municipalities of the province of Cuenca, Castilla-La Mancha region (Spain), were invited to be part of the study. One school refused to participate stating that the study could mean excessive additional work for teachers. After acceptance of the schools to participate in the study, all schools were randomly allocated using the statistical package StatsDirect as follows: intervention MOVI-da10-Enriched! focused on enriched PA breaks while learning or reinforcing the academic contents of different subjects (*n* = 3), intervention MOVI-da10-Standard! focused on PA breaks only (*n* = 2) or the control group (n = 3) (Fig. 1).

### Recruitment and allocation

First, investigators visited each school to provide information about the aim and methods of the study, and to obtain the consent of the School’s Director and the School Board at the same time. Subsequently, parents were invited by a letter to a meeting for explain the procedures and objectives of this study. After that, parents signed informed consent to approve their children’s participation in the study. Children’s results were sent to the parents or tutors by a letter in each measuring process. The team doctors gave recommendations to families when there were anomalous values from the children.

### Inclusion and exclusion criteria

To participate in the project, the schools had to have at least two full classrooms of the third year of preschool (4 to 6 years old). Additionally, the study was approved by the each School Board at the start and the end of the academic year. Children were excluded when: (i) presenting Spanish learning problems; (ii) teachers, parents or tutors reported any children’s serious physical or mental disorders, which could prevent participation in the activities of the study; and (iii) pediatricians informed any schoolchildren’s chronic disorder, such as heart diseases, diabetes or asthma that could prevent participation in the physical activities of the program. Additionally, the collaboration of a family member to fill out a questionnaire about the child’s lifestyle was required.

### Ethical and legal aspects

The Clinical Research Ethics Committee of the “Virgen de la Luz” Hospital of Cuenca approved the study protocol. The MOVI-da10! trial is registered in ClinicalTrials.gov (NCT03236363).

### Interventions

#### MOVI-da10! programs: description

The design of the two interventions of the MOVI-da10! is based on the socio-ecological model [22], in which behaviour is understood as the interaction between personal and environmental factors that determine behaviours. The intervention period lasted one academic year (from October 2017 to May 2018), during which children in the two intervention groups received on average two breaks/day lasting 10 min in the classroom every school day. The breaks did not require specific material.

#### - MOVI-da10-enriched!

**Fig. 2:**
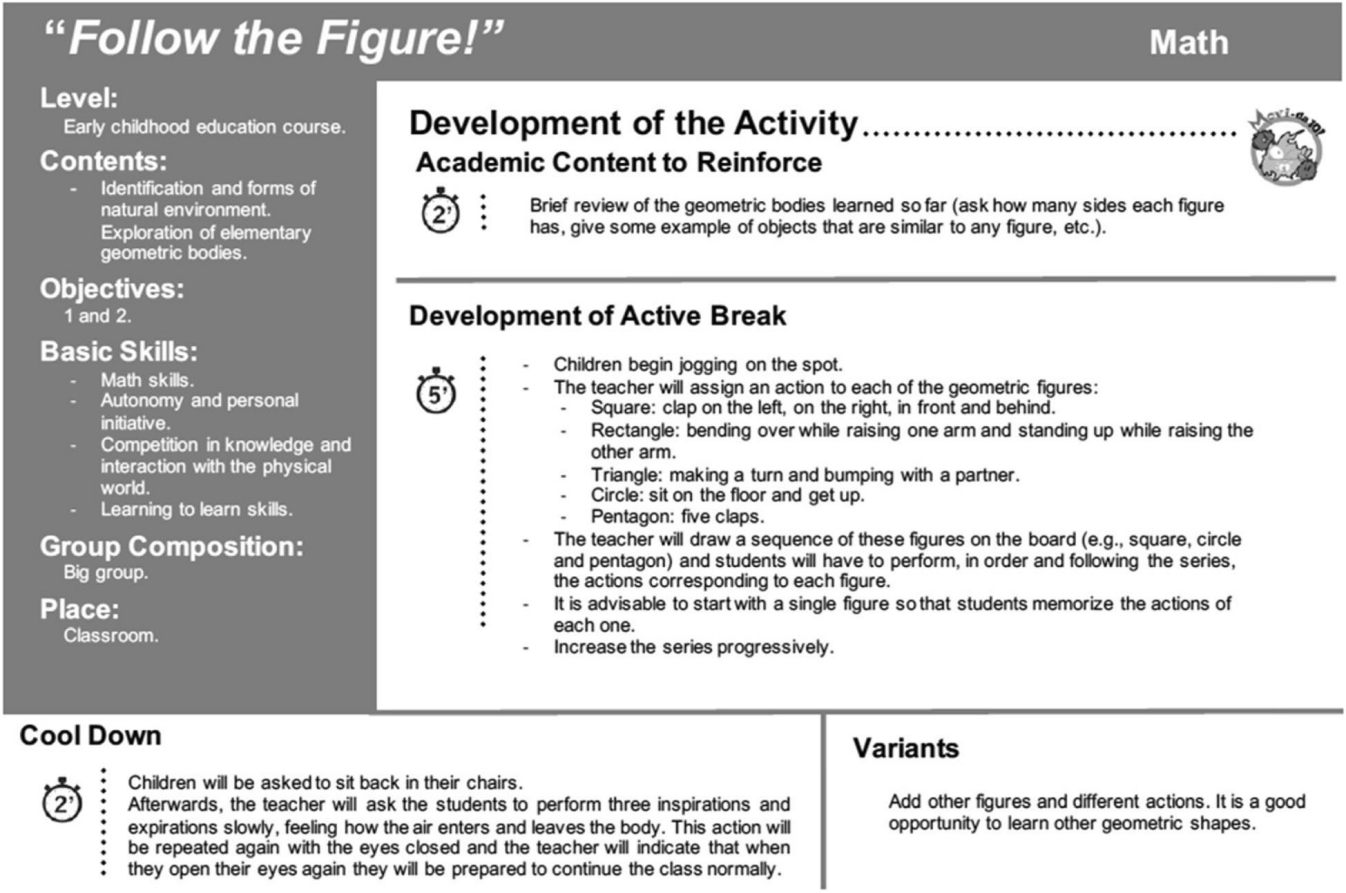
MOVI-da10-Enriched! Session type

Sixty PA breaks were designed to develop curricular contents for children aged 4 to 6 years old including counting, numbers, basic mathematical operations (such as addition and subtraction), the body, or letters. Each break developed a specific curricular content through coordination exercises (bilateral body coordination) and basic motor skills (balance, jumps, displacements and handling of objects) of high cognitive demand. The enriched PA breaks consisted of 1 to 2 min to reinforce the curricular content, 6 min of moderate intensity PA (3 to 4 metabolic equivalents of task [METs], heart rate < 150 bpm), and 1 to 2 min to perform cool down exercises and return to regular academic activities. Figure 2 shows an example of a MOVI-da10-Enriched! PA break .

#### - MOVI-da10-standard!

**Fig. 3:**
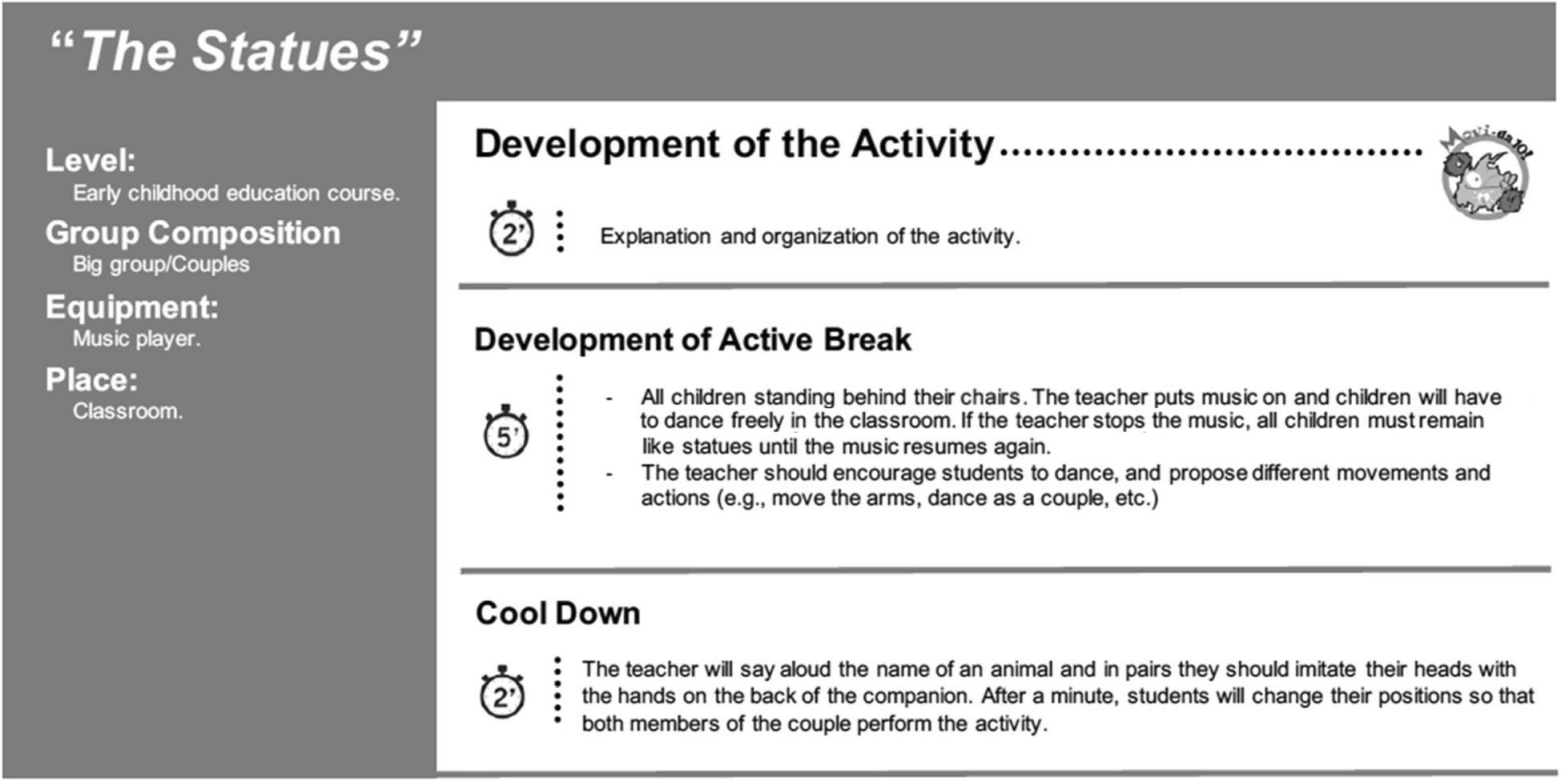
MOVI-da10-Standard! Session type

Sixty breaks of PA were designed that include simple games or low cognitive demand activities that did not aim to reinforce curricular contents, such as dancing following the rhythm of a musical instrument, moving around the class and following the teacher’s instructions (running, touching the floor and jumping, getting on the chair), etc. The standardized PA breaks were structured as: 1 to 2 min to explain the activity, 6 min of moderate-vigorous intensity PA (5 to 6 METs, heart rate ≥ 150 bpm), and 2 min to perform cool down exercises and return to regular academic activities. Figure 3 shows an example of a MOVI-da10-Standard! PA break.

#### MOVI-da10! programs: organization and functioning

The Movi-da10 programs were designed by two sport sciences and PA graduates, and applied by teachers in the classroom. Teachers were blinded to the schools group assignment. Teachers were skilled in the MOVI-da10! programs through a 1 day training session in order to standardize the implementation of the program. Teachers received new activities by email quarterly. In addition, these emails encouraged them to continue with the program and reminded them about the importance of carrying out the intervention. To respect the autonomy of the teachers and facilitate the implementation of the interventions within the classroom, teachers could choose the content and the time of the day to implement the PA breaks. Team members recommended using PA breaks, mainly when children: i) spend a lot of time sitting, ii) do not pay attention in the classroom or iii) feel tired of the academic contents. Furthermore, to facilitate the work of teachers, the research team provided a school schedule in each class to record the time of the day and number of PA breaks made each day.

#### MOVI-da10! programs: process evaluation

To ensure the desired intensity in each intervention, before starting the programs, a pilot study was carried out using heart rate monitors (Polar A300 and HR sensor H7). After 16 weeks of the program, researchers visited the participating schools to confirm the intensity of the breaks by placing heart rate monitors on randomly selected children. An email address and a telephone number were available for teachers over the academic year. Monthly contacts with teachers were also held by phone and e-mail to obtaininformation about the children’s motivation and the development of the program. Additionally, two meetings were conducted with teachers, at baseline and 4 months later. After 2 months of the program’s implementation, the teachers answered a questionnaire where they were asked on average how many PA breaks were implemented weekly, the attitude and behavior of the children during PA breaks, anything that could be improved, and what was their motivation and main barriers to implement the PA breaks (please see Table 1). To encourage adherence to the MOVI-da10! programs, children were given positive reinforcements and received small gifts showing the logo of the study with the mascot (T-shirt, stickers, etc.).

**Table 1:** Questionnaire of evaluation and follow-up of the MOVI-da10! Program for teachers

Questions	Answers			
	Always	Sometimes	Rarely	Never
1. The children show interest in participating during activities				
2. Activities have improved group cohesion in class				
3. Children have fun with the activities that are developed				
4. Children participate with interest in activities				
5. Activities are useful to review and reinforce the curriculum contents already seen in class^a^				
6. Children are more attentive in the following task after carrying out activities				
7. Children are more relaxed in the following task after carrying out activities				
8. Children are very physically tired after doing activities				
9. Children are very cognitively tired after doing activities				
10. Children perform activities at moderate-high physical activity intensity (these involve a considerable increase in heart rate, even reaching fatigue)^a^				
11. hildren perform activities at moderate physical activity intensity (these involve a considerable increase in heart rate, but without reaching fatigue)^b^				
12. Proposed activities are appropriate for the children’s level of development				
13. The proposed activities carried out are boring				
14. After the activities, I have difficulties in continuing the class normally				
15. The proposed activities carried out lasted more than 10 min				
16. The proposed activities are limited by space within the classroom				
17. I have had too many difficulties in carrying out the proposed activities				
18. I think that, after finishing the program, these activities will remain as a usual methodology in the classroom				
19. When I have had some problem, I have been treated satisfactorily by the coordination of the study				
20. In general, I am satisfied with the development of the MOVI-da10! program				
21. Normally, how many active breaks do you do weekly? (write the number)				

^a^Question only asked for the MOVI-da10-Enriched! Program

^b^Question only asked for the MOVI-da10-Standards! Program

Children in both the intervention groups (IGs) and the control group (CG) received the standard physical education curriculum (1 h/week of psychomotor activities for third year of preschool children with PA levels at low-to-moderate intensity) as it is compulsory in Spain. We recommended the teachers of the CG to avoid implementing any modifications in their methodology during the duration of the study, with the promise of the research team to share and explain the MOVI-da10 materials once the interventions were finished.

## Outcome measures

Baseline and post-intervention outcome variables were evaluated in all groups twice, at the beginning (September 2017) and at the end (June 2018) of the school year. The measurements were completed at school by trained investigators to minimize inter-observer variability and blinded to the group in which schoolchildren were allocated.

### Primary outcome measures

#### Anthropometry variables

Weight was measured twice (Seca® 861 scales) with the children in lightweight clothing and barefoot. Children’s height was measured twice by a stadiometer (Seca® 222). Children were barefoot, upright and with the sagittal midline touching the wall.

#### Body composition

Body mass index of children was calculated as weight divided by height square (kg/m^2^). Waist circumference was measured twice at the end of the expiration in the middle point between iliac crest and costal margin when the child was upright using a meter tape. Tanita BC-418 MA bioimpedance analysis system was used to measure twice body fat-free and fat status of the children. The measurement was conducted in the morning after urination and resting 15 min, without footwear and fasting under temperature and humidity control.

#### Blood pressure

The arterial systolic and diastolic blood pressure were evaluated twice (with an interval of 5 min between measurements) using an an OMRON-M5-I automatic tensiometer (Omron Healthcare UK Ltd.) where the child was seated, in a quiet environment, with the right arm semi-flexed at the level of the heart. Two readings were obtained and their mean was considered for analyses.

#### Physical fitness

Was measured using the PREFIT battery [23, 24] as follows:

*Cardiorespiratory fitness* was evaluated by the Course Navette test (20-min shuttle run test), validated to evalue the maximal aerobic capacity in preschoolers [25]. Children had to run between two lines 20 m apart, while keeping rhythm with audio signals emitted from a prerecorded CD and they were encouraged to run as much as possible throughout the course of the test. The beginning speed was 6.5 km h-1, and increased by 0.5 kmh-1 min-1 (one min equals one stage). Maximal oxygen intake was calculated by using the preschool-adapted 20-m shuttle-run (PREFIT) formula [26].

*Muscle strength* was measured using dynamometry (Takey®, TKK 5401 Grip-D) and the standing long jump that measured both upper and lower explosive body strength. In the first one, participants gradually and continuously tightened for at least 2 seg using the optimal grip measure doing the right and left hands in turn. Two attempts were made with both hands with a short resting time between them, and the maximum score in kilograms for each hand was recorded. In the second one, children had to jump horizontally to achieve maximum distance from a starting position immediately behind a line and not touching it, standing with feet approximately shoulder width apart. The best of three attempts was recorded in centimeters.

*Speed-agility* was measuresed using the 4 × 10 shuttle run test. Children had to run four times as fast as they could between two lines 10 m apart. Two measurements was conducted with an interval of 5 min, and only the best attempt was used for analyses. A lower score indicates better speed-agility.

*Flexibility* was evaluated by the sit-and-reach test, which measures the maximum distance schoolchildren could reach with their fingertips by flexing the trunk without bending the knees. Children had three attempts [27].

### Cognition

Two core executive functions were measurement using two validated tests of the NIH toolbox battery [28] for the population aged between 3 and 85 years old. The Flanker Inhibitory Control and Attention test was used to measure inhibition/attention, this version includes a practice block of 4 trials, that children passed if they completed at least 3 trials. If so, a 20-trials block was presented consisting of a pseudorandom sequence of congruent and incongruent trials. Reaction time and accuracy percentage on incongruent and congruent trials were used as outcome of inhibition/attention; moreover, raw scores were attained [29]. Cognitive flexibility was assessed by the Stort dimensional change card test. Through this tool, schoolchildren should classify a series of bivalent test cards according to different dimensions of “color” or “shape”. First, a block of 4 trials was performed, from which participants had to pass three to get to the mixed 30-trial block randomly. Reaction time and accuracy percentage on pre-switch and post-switch trials were used as outcome of cognitive flexibility; in addition, raw scores were obtained [29].

### Secondary outcomes measures

#### Motor competence

Was assessed through 5 tests of the Movement Assessment Battery for Children, 2nd edition (MABC-2) [30] that evaluate gross motor skills. For children aged 4 to 6 years old these include: catching beanbags with two hands and throwing beanbags onto mats for ‘aiming and catching’, and balancing on one leg, walking with the heels raised and jumping on mats for ‘static and dynamic balance’. Each test received a raw score (time-related scored in seconds or error-related scored by number of ‘good’ attempts), which were converted into scaled scores based on age and the normative reference values proposed by the MABC-2 manual. A higher score indicated better motor competence.

#### Health-related quality of life

Was evaluated by Kiddy-Kindl questionnaire, which has been validated for Spanish children aged 4 to 7 years old. This questionnaire is a generic health related quality of life instrument for children, which contains 12 questions divided into six dimensions (self-esteem, emotional, physical, school, family and friends). The children’s version was administered by interview, while the parents were offered a self-administered version [31].

#### Physical activity

Was measured in a subsample of 500 randomly selected schoolchildren belonging to the IGs and the CG. PA was measured using GENEActive accelerometers (ActivInsights) for 7 consecutive days (including nights), with a fixed frequency of 30.0 Hz to record the data raw of acceleration measured in “g” for each movement axis (x, y and, z). The data were expressed in units of milli-g (1000 mg = 1 g = 9.81 m / s2) [32]. A valid measurement was considered for reports of at least 5 days, including one weekend day.

### Confounding variables

The following confounding variables were evaluated: sex, age, birth weight, gestational age, familiar socioeconomic status, weight and height of parents (self-reported), breastfeeding, food consumption, area (urban or rural), and origin (native or foreign).

#### Food consumption

Food consumption was estimated using the Spanish version of the Children’s Eating Habits Questionnaire, validated for children from 2 to 9 years old. This questionnaire was fulfilled by the parents [33].

#### Mothers’ breastfeeding

Parents were asked what type of feeding had been chosen during their children’s first 24 months of life. In each month, systematically, mothers could indicate if their children had been fed by breast milk, artificial milk, both and/or complementary feeding. Mothers were able to mark one or several options from all those indicated. In this way, it was possible to categorize breastfeeding into exclusive breastfeeding, mixed breastfeeding and artificial lactation, indicating the duration of each.

#### Familiar socioeconomic status

The familiar socioeconomic status was measured as in previous MOVI studies [34]. A questionnaire self-reported by the father and mother about their education and occupation was used. The education of the parents was categorized into three categories: primary education (functionally illiterate, without any studies or those who had not completed primary education), middle education (primary education and high school or secondary education or ‘Bachillerato’) and university education (university degree or Ph.D.). The occupation of the parents was categorized as: (i) supervisor, manager or freelance with ten employees or more, (ii) supervisor, manager or freelance with fewer than ten employees, (iii) freelance with no staff, (iv) unqualified staff and unskilled workers, and (v) household chores, unemployed or others. After, an index of familiar socioeconomic status was calculated using both indicators (parents’s education and occupation). This index allows the establishment of five categories of familiar socioeconomic status: lower, upper lower, lower middle, upper middle and upper [35].

## Data analysis and management

### Sample size

The sample size for a three-arm cluster randomized trial was calculated using the WebPower software and selecting the omnibus test option considering an average of 50 children by school (cluster), a statistical power of 0.8, a significance level of 0.05, and an effect size of 0.5 on cognitive performance (selective attention using Das and Naglieri-Cognitive Assesment System) on the basis of a previous pilot study, according to the methods for this type of study proposed by Donner and Klark [36]. Taking into account these assumptions, the estimated sample size was 9 schools (clusters), three per arm. It should be noted that the number of subjects per cluster considers that the average number of students/classroom is 25, and schools with at least two classrooms were considered.

### Statistical analysis

The data of participants will be entered into a database by two independent researchers at the end of the measurements. Blinding on the handling of data will be assured by separating measurement values from the participant’s personal identification information. The statistical analysis will be performed in three steps. First, the effectiveness of the randomization processes will be evaluated through exploring the normal distribution of the variables and outliers using graphical procedures and the Kolmogorov Smirnov test. Social comparisons will be established between the CG and the two IGs, and subsequently, if appropriate, between MOVI-da10-Enriched! (IG-A) and MOVI-da10-Standardized! (IG-B).

Second, mixed regression models [37] will be used to evaluate the differences between the baseline and final variable measurements considering three models: Model 1 (1 = IG-A and 0 = CG), Model 2 (2 = IG-B and 0 = CG) and, if there is significant improvement in the cognitive performance of the IGs versus CG in the first two models, a third model (Model 3) will be made (1 = IG-A and 2 = IG-B). For these analyses, each outcome variable will be considered as independent, and the intervention as a fixed effect. Furthermore, the analyses will be adjusted for baseline data, age, sex and school (cluster). The results will be expressed in absolute differences in the changes in the variables between the baseline and the final measurements (95% confidence interval [95% CI]). When the dependent variable is prevalence of overweight/obesity, odds ratios and their 95% CI will be estimated.

In the third step, a comparison will be made independently of the IG-A and the IG-B against the CG using the propensity score statistical method in order to consider the potential imbalance of the covariates in the basal measurements among the clusters. The propensity score estimates the effect of the intervention using a model of causal inference, explaining what would have happened if all the subjects of the IGs and the CG had the same characteristics at the beginning of the study. Each subject will be matched to a subject with similar characteristics using a caliper of 0.40 using the STATA psmatch2 command. The propensity analysis generates a standardized coefficient with its corresponding confidence interval, in such a way that the higher standardized coefficient represents the more effective intervention as compared with the control one, whenever the confidence intervals of the coefficients do not overlap, the differences between coefficients are not significant.

All the analyses will be carried out considering intention to treat, keeping children in the group they were originally allocated, regardless of the number of PA breaks carried out, and taking into consideration the CONSORT guidelines for cluster RCTs. [38].

Results will be considered statistically significant at *p* < 0.05. The analyses will be performed using STATA 15 version.

## Discussion

Schools could be the ideal setting to help children meet PA guidelines. Physical Education classes and recesses have traditionnally been the periods of time in the school day in which students could accumulate PA, but nowadays, taking into account the decrease in the PA levels of students, and the rates of overweight and obesity, it seems necessary to explore new alternatives to increase PA time during the school day. Although numerous benefits related to both health and education of PA integrated in the classroom are promising, the results are not yet conclusive, and the results of the last reviews and meta-analyses highlight the need for more interventions in this area [15, 16, 39].

To our knowledge, MOVI-da10! is the first RCT addressing, in preschool children, the comparison of the effectiveness of two PA programs conducted in the classroom (enriched PA integrated into the academic curriculum and PA breaks only) on improving adiposity, executive function and motor competence. The secondary aim is to examine the impact of the programs on PA and improving health related quality of life or motor competence.

Some strengths of this study are that: i) it is conducted inside the school, which guarantees scaling up, since it is accessible to all students regardless of race, sex, ethnicity or family socioeconomic status. Additionally, no other institution has as much influence on children during the first years of their lives; furthermore, the mandatory nature of formal education could increase the effectiveness of this type of intervention [20]; ii) it has been shown that increasing opportunities for students to be physically active during the school day does not compromise academic performance [40]; iii) the program includes planned and structured activities, which are easily reproducible, but respects the teacher’s autonomy since they can decide what activity to do each day and at what time of the day, this fact could increase the acceptability of the program by teachers; iv) this study uses a standardized measure of cognitive function with established validity and reliability to be able to make comparisons with other studies; v) the use of an objective measure of PA that will allow knowing the exact intensity of PA breaks and the effect of MOVI-da10! in increasing the PA of children.

Some limitations should be mentioned. Although this is an RCT, children, parents and teachers could not be blinded regarding the allocation group and some bias could be derived from this fact. This bias was reduced by using a cluster randomization design. Additionally, during interventions, teachers may not implement PA breaks exactly as the researcher intended, thus adding some variability. Teachers might use PA breaks less frequently than planned, thus shortening duration or reducing intensity. This bias was minimized by training teachers, standardizing the program (providing pre-packaged PA breaks) and direct contact with teachers.

## Abbreviations

CG: Control group
CI: Confidence interval
IG: Intervention group
IG-A: MOVI-da10-Enriched! Intervention group
IG-B: MOVI-da10-Standardized! Intervention group
METs: Metabolic equivalents of task
PA: Physical activity
RCT: Cluster-randomized control trial
